# Chronic myelomonocytic leukemia (CMML)-0 with pleural effusion as first manifestation: A case report

**DOI:** 10.1097/MD.0000000000023030

**Published:** 2020-10-30

**Authors:** Lianbo Hu, Bingrong Zheng, Lijuan Fu, Meiwei Hu

**Affiliations:** Department of Hematology, The Second Affiliated Hospital of Zhejiang Chinese Medical University, Hangzhou, Zhejiang, China.

**Keywords:** azacitidine, chronic myelomonocytic leukemia, pleural effusion

## Abstract

**Rationale:**

Extramedullary invasion of chronic myelomonocytic leukemia (CMML) usually occurs in the liver, spleen, and lymph nodes, while the pleural infiltration of CMML is rare. The presence of pleural effusion is usually associated with uncontrolled leukocytosis and increased monocytes.

**Patient concerns:**

Here we reported a rare case of CMML-0 with pleural effusion as the first manifestation in a 44-year-old woman. The pleural effusion was caused by blasts infiltration confirmed by the flow cytometer and the pleural biopsy.

**Diagnoses:**

CMML with pleural invasion.

**Interventions:**

The patient was treated with azacitidine 75 mg/m^2^ d for 2 cycles, followed by daily oral intake of hydroxyurea (500 mg/d).

**Outcomes:**

Pleural effusion was resolved and chest pain was relieved.

**Lessons:**

The current case indicated that leukemic infiltration into pleura could occur despite mild leukocytes, while demethylation may be an effective therapy.

## 1 Introduction

Chronic myelomonocytic leukemia (CMML) is characterized by myelodysplastic syndrome and myelo-proliferative tumor.^[1]^ The incidence of CMML is low and the mean age of diagnosis is 65 to 70 years.^[2]^ Extramedullary leukemic infiltration, which can be observed in CMML, is most commonly found in the liver and spleen, but can sometimes invade the skin, gingiva, and other areas during disease progression.^[3]^ Serous effusion including pericardial effusion, pleural effusion, and ascites, is a rare complication of CMML.^[4]^ Pleural effusion is usually caused by leukemic infiltration into the pleura and may also be associated with extramedullary hematopoiesis and immune responses. Pleural effusion is closely related to the transformation from CMML to acute leukemia. In the present study, we report a rare case of pleural effusion in a patient with CMML-0 and slight monocytosis as the first clinical manifestation.

## 2 Case presentation

**Figure 1:**
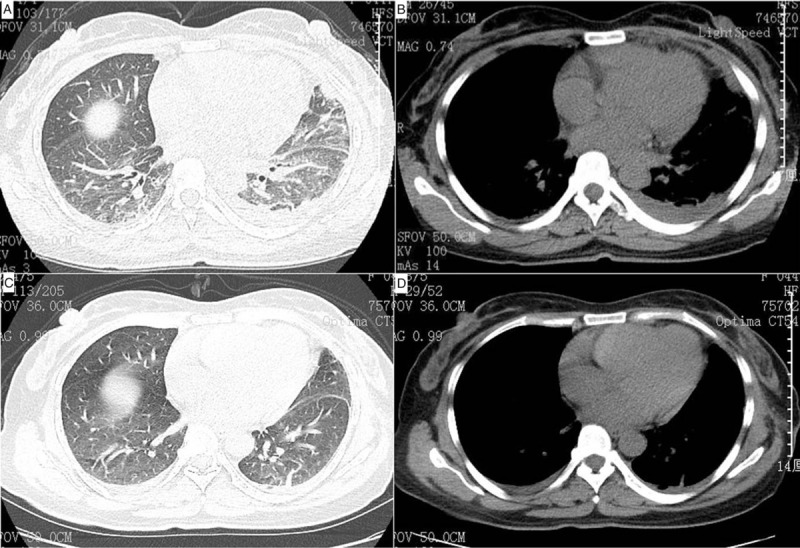
(A and B) Chest computed tomography (CT) showed pulmonary infection, bilateral pleural effusions, and left pleural hypertrophy. (C and D) CT showed the pleural effusion was almost absorbed after 2 courses of azacitidine treatment.

**Figure 2:**
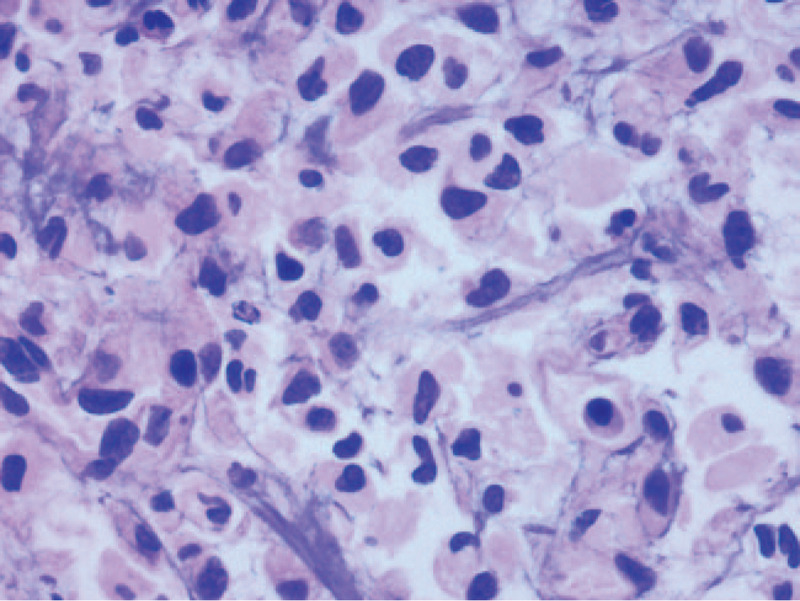
Pleural biopsy showed heterotypic mononuclear cell infiltration in fibrous adipose tissue, with a few lymphocytes, plasma cells and neutrophils, H&E stain 400×. H&E = hematoxylin and eosin.

A 44-year-old woman was admitted to our hospital in July 2019 due to cough, sputum production, and chest pain lasting for 1 week. Chest computed tomography showed pulmonary infection, bilateral pleural effusions, and left pleural hypertrophy (Fig. 1). The initial complete blood count revealed: white blood cell (WBC) count 21.5 × 10^9^/L (22.7% monocytes, 65% neutrophils, 8.1% lymphocytes), hemoglobin 132 g/L, and platelet 233 × 10^9^/L. C-reactive protein level was 13.1 mg/L (0–10 mg/L). The patient received antibiotics; however, her clinical symptoms did not improve, and the WBC count increased to 22.3 × 10^9^/L with 26.9% monocytes, while no blasts were observed. The pleural effusion was bloody. Thoracocentesis indicated exudative effusion with 47 g/dL total protein, 277 U/L lactate dehydrogenase, and 7.37 mg/dL glucose concentration. The Rivalta test was positive, with a nucleated cell count of 12,800/μL, red blood cell count of 4800/μL (the ratio of red blood cells to nucleated cells was obviously lower than that of the peripheral blood), and 86% of macrophages. The cytology revealed no malignant cells, but 0.7% of blasts were detected by the flow cytometer. Microbial screening culture identified no organisms. The chest pain obviously improved after thoracocentesis; nevertheless, the severe chest pain recurred after 5 days of thoracocentesis. Subsequently, the pleural biopsy was performed under thoracoscopy. Extensive pleural adhesions were found in the patient. Postoperative histopathology confirmed heterotypic mononuclear cell infiltration (Fig. 2) in fibrous adipose tissue, with a few lymphocytes, plasma cells and neutrophils. Immunohistochemistry (IHC) were: CD68+, CD20 B cells+, CD79a B cells+, CD3 T cells+, CD43 T cells+, CD10−, S-100−, CR mesothelial cells+, D2–40−, Ki67 hotspot area about 20%+. Bone marrow aspiration revealed the increase in blasts (3%), and 2.9% blasts that had the same immunophenotype as the pleural effusion with abnormally expressed CD56 on granulocytes by flow cytometer. The bone marrow biopsy specimen showed extremely active hyperplasia, megalokaryocyte with a single round nucleus, and no reticulin fibrosis. The G-banding analysis revealed normal karyotype (46, XX); BCR/ABL fusion gene and other genes were not detected by the PCR assay, while ASXL1, SETBP1, BRAF, SETD2 mutations were observed.

Based on these results, the patient was diagnosed with CMML-0 according to WHO 2016. She was treated with azacitidine 75 mg/m^2^ d for 2 cycles, followed by daily oral intake of hydroxyurea (500 mg/d). Within 2 weeks, the chest pain was relieved. After 2 courses of azacitidine treatment, pleural effusion was almost absorbed (Fig. 1). The patient is currently free of symptoms (chest pain and pleural effusion), and the monocytosis has been under control for 6 months.

## 3 Discussion

Here we report a rare case of CMML with pleural effusion as the initial manifestation. This case was diagnosed as CMML-M0 according to the WHO diagnostic criteria from 2016.^[1]^ Our patient had pleural effusion as the first symptom that led to the diagnosis of CMML-0 stage. It has been reported that 1 out of 60 CMML patients (1.7%) has serosal effusion at the time of diagnosis.^[5]^ So far, more than 10 cases of CMML with pleural effusion have been found since the 1990s (Table 1). Pleural effusion occurs during the whole clinical process of CMML and is usually accompanied by a high or rapid increase in leukocyte and monocyte count. Yet, 1 patient with CMML developed pleural effusion during the decline of WBC and monocytes.^[13]^ It is important to emphasize that it is rare for patients with CMML to have mild leukocytosis accompanied by pleural effusion as the first clinical sign.

**Table 1.**
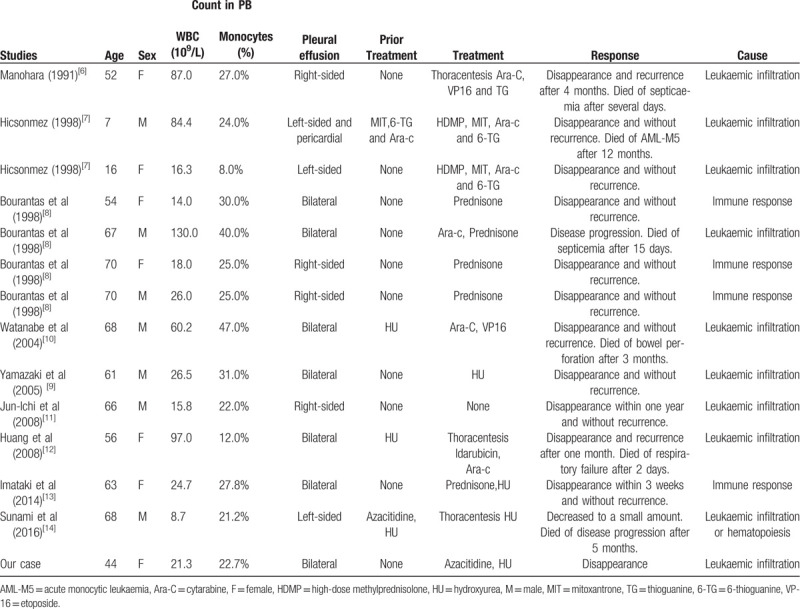
Literature reports on CMML presenting with pleural effusion.

There are several possible mechanisms for the development of pleural effusion in CMML patients, the first one being a non-malignant cause, such as infection. This mechanism was excluded in our patient by the identification of microorganisms. The second mechanism is the infiltration of leukemia into the pleura, which usually occurs at the time of transition to the acute leukemia stage or before.^[6–8,12]^ Pleural effusion analysis of these cases revealed different stages of monocytes and leukemic cells. Cases of pleural invasion of leukemia without leukemia transformation are relatively rare.^[11,14]^ The patient's bone marrow blasts had the same immunophenotype as the pleural effusion, but there were no blasts in peripheral blood. Pleural biopsy pathology also suggested atypical mononuclear cell invasion. Although there was no crisis of leukemia transformation, it was still considered that the patient's pleural effusion was caused by leukemia cells invading the pleura. The third mechanism includes a variety of causes leading to bleeding into the pleural cavity. At this point, the proportion of red blood cells and nucleated cells in the blood should be similar to the effusion. However, the ratio of red blood cells to nucleated cells in the pleural effusion was obviously lower than that of the peripheral blood. The presence of 86% macrophages in the pleural effusion also suggested that the patient's pleural effusion was non-bleeding. The fourth possible mechanism is pleural extramedullary hematopoiesis. Myelofibrosis is often associated with extramedullary hematopoiesis. CMML is also a proliferative tumor of bone marrow. Extramedullary hematopoietic sites generally include the liver, spleen, and lymph node. The pleura can also be the site of extramedullary hematopoiesis, while pleural effusion may also be caused by extramedullary hematopoiesis. Extramedullary hematopoiesis implies hematopoiesis of erythroid, myeloid, and megakaryocytes, and a dominant lineage, which may appear.^[14]^ The patient's bone marrow biopsy confirmed no myelofibrosis, and pleural effusion and pleural biopsy showed no other hematopoietic cells. There is the fifth mechanism that needs to take into account the immune response. The cause of pleural effusion in CMML is an immune response to bacteria or viruses.^[5]^ Bourantas *et al* reported that in 4 cases of CMML with pleural effusion, only 1 occurred due to CMML cell infiltration and the remaining 3 were immune responses.^[8]^ Monocytes are hyperproliferated in the immune response, which needs to be determined by flow cytometry. An immune response did not cause pleural effusion in our patient according to the immunophenotypes.

For the treatment of CMML with pleural effusion, there is no uniform standard due to the rare incidence of the condition. Patients with pleural effusion are reported (Table 1) for treatment with hydroxyurea, etoposide (VP16), idabil, and cytarabine (Ara-c). At present, allogeneic hematopoietic stem cell transplantation is the only possible method to cure CMML. However, the patient did not have a suitable bone marrow match. Over recent years, demethylated drugs have been used to treat CMML. Methylation can lead to mutations in related genes, including TET2, UTX, EZH2, and DNMT3A. The use of demethylated drugs can prolong the median overall survival time in patients.^[15]^ Our patient chose azacytarine for treatment. After 2 courses of azacitidine treatment, pleural effusion was almost absorbed, and chest pain relieved. The patient refused further use of azacytarine, although it was well tolerated. She was transferred to hydroxyurea for maintenance therapy. After half a year of outpatient follow-up, the patient's peripheral blood leucocytes were stable with 15 × 10^9^/L, and the proportion of monocytes was about 30%. The patient's pleural effusion did not recur. Little information is available on the treatment of CMML with extramedullary invasion. Our patient was treated with azacitidine and the pleural effusion was relieved. Azacitidine may relieve extramedullary pleural invasion in CMML. Jun-Ichi *et al* proposed that pleural effusion without respiratory failure might not necessarily require an active treatment in CMML without the transformation of acute leukemia.^[10]^ Future studies should investigate CMML with pleural effusion so as to provide more data for appropriate treatment strategies.

## 4 Conclusion

The invasion of the pleura has been rarely documented in CMML, especially not at the stage of blast proliferation or transformation to leukemia. In this case of CMML with pleural metastases, demethylation, and maintenance hydroxyurea treatments showed to be effective. More cases are required to confirm whether azacitidine is beneficial for CMML with extramedullary invasion, especially pleura.

## Author contributions

**Conceptualization:** Lijuan Fu.

**Formal analysis:** Meiwei Hu.

**Project administration:** Meiwei Hu.

**Writing – original draft:** Lianbo Hu.

**Writing – review & editing:** Bingrong Zheng.
